# Chronifiziertes malignes Glaukom nach Kataraktoperation

**DOI:** 10.1007/s00347-020-01088-4

**Published:** 2020-04-01

**Authors:** Cornelius Wiedenmann, Stefaniya Boneva, Alexandra Anton, Thomas Reinhard, Jan Lübke

**Affiliations:** Klinik für Augenheilkunde, Universitätsklinikum Freiburg, Universitätsklinikum Freiburg, Killianstr. 5, 79106 Freiburg, Deutschland

**Keywords:** Kammerwasser-Fehlleitung, Ziliolentikulärer Block, Kataraktchirurgie, Myopisierung, Augeninnendruckerhöhung, Aqueous misdirection, Ciliolenticular block, Cataract surgery, Myopia, Increased intraocular pressure

## Abstract

Das maligne Glaukom mehrere Jahre nach Kataraktoperation stellt eine sehr seltene Spätkomplikation dar. Eine zunehmende Myopisierung kann einen frühen Hinweis auf das Entstehen geben. Eine chirurgische Intervention ist oft unumgänglich. Die Druckeinstellung kann wie in unserem Fall aber auch konservativ gelingen. Es kann daher der Situation angepasst und individuell entschieden werden, wie invasiv die Behandlung erfolgen muss und ob eine Vitrektomie zwingend notwendig ist.

## Abstract (en)

The occurrence of malignant glaucoma several years after cataract surgery is a very rare delayed complication. An increasing myopic shift can be an early hint for the formation. A surgical intervention is often inevitable; however, intraocular pressure can also be successfully managed with conservative treatment as in the case described. Therefore, treatment should be adapted to the situation and individually decided how invasive the treatment must be and whether a vitrectomy is absolutely necessary.

## Anamnese

Eine 83-jährige Patientin stellte sich Ende 2018 laut Überweisung wegen Tensioentgleisung und neu diagnostiziertem Gesichtsfeldausfall an ihrem linken Auge (LA) in unserer augenärztlichen Ambulanz vor. Die Patientin schilderte, links wie durch einen Schleier zu sehen, welcher schleichend aufgetreten sei und langsam zugenommen habe. Anamnestisch sei das LA seit der Kataraktoperation an beiden Augen 2011 nie so gut gewesen wie das rechte Auge (RA). Weitere ophthalmologische Vordiagnosen bestünden nicht. Ein Trauma sei nicht erinnerlich.

## Befund

**Figure 1:**
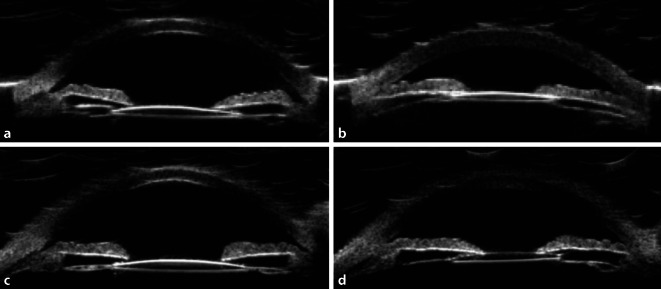

**Figure 2:**
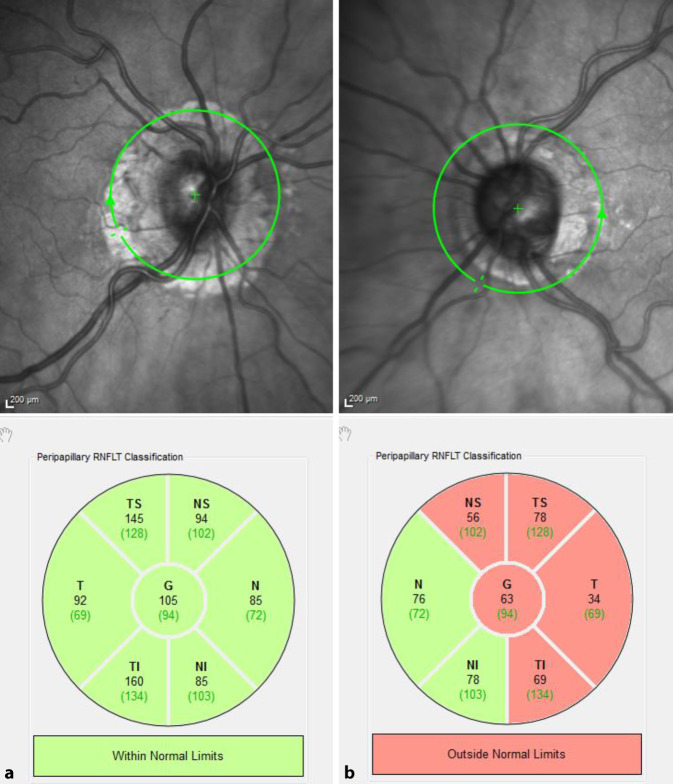

**Figure 3:**
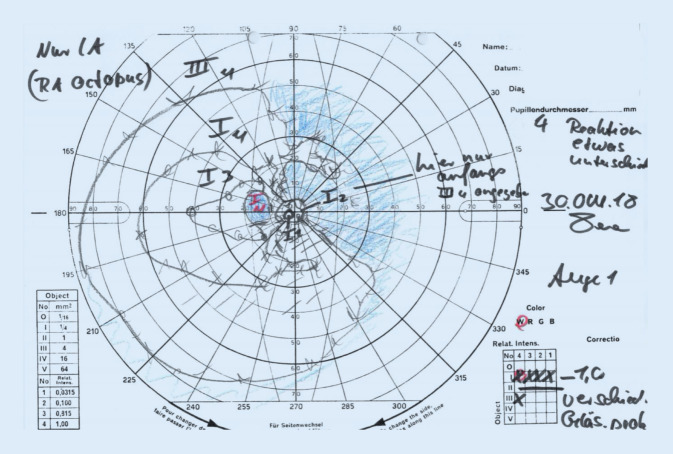

Bei Erstvorstellung lag der Visus am LA bei 0,1, am RA bei 0,8. Im Vergleich zu den Refraktionswerten der eigenen Brille (RA: −0,5 −1 115°, LA: −2 −0,25 114°) zeigte sich in der durchgeführten Autorefraktion eine deutliche Abweichung am linken Auge (RA: −0,5 −0,75 105°, LA: −3,75 −0,75 55°). Der intraokulare Druck (IOD) am LA war auf 31 mm Hg erhöht, am RA mit 15 mm Hg normwertig. Auffällig am Vorderabschnittsbefund war der deutliche Seitenunterschied der Vorderkammertiefe (VKT) (Abb. 1a, b). Eine Pseudoexfoliation der Linse (PEX) zeigte sich an keinem der Augen. Auch eine Pseudophakodonesis lag nicht vor. Die Intraokularlinsen (IOL) lagen auf beiden Seiten regelrecht im Kapselsack. Die Papille links war temporal randständig exkaviert. Eine Spectralis-OCT (optische Kohärenztomographie, Heidelberg Engineering GmbH, Heidelberg, Deutschland) mit RNFL(retinale Nervenfaserschicht)-Darstellung wies am linken Auge eine von nasal-superior über temporal bis temporal-inferior reichende Verdünnung des Nervenfaserrandsaums und eine auf 63 µm reduzierte Verminderung der mittleren Nervenfaserschichtdicke auf (Abb. 2). Links zeigte sich in der Goldmann-Perimetrie (Haag-Streit Deutschland, Wedel, Deutschland) nasal oben und im oberen Bjerrum-Bereich ein bis nach zentral und über die Quadrantengrenzen oben und nasal reichender Ausfall aller Lichtmarken (Abb. 3). Eine Vermessung des vorderen Augenabschnitts beider Augen mittels Pentacam (OCULUS Optikgeräte GmbH, Wetzlar, Deutschland) ergab die in Tab. 1 aufgeführten Werte für Vorderkammertiefe (VKT), Kammervolumen (KV) und Kammerwinkel (KW).

**Table 1:** 

Datum	RA					LA				
	T in mm Hg	KV in mm^3^	VKT in mm	KW	Sphäre in dpt	T in mm Hg	KV in mm^3^	VKT in mm	KW	Sphäre in dpt
30.10.2018	14	150	3,34	38,8°	−0,5	30	83	2,12	20,0°	−3,75
02.11.2018	14	161	3,56	45,6°	−0,5	9	111	2,68	33,6°	−2,75
06.11.2018	11	157	3,51	44,0°	+0,25	13	117	2,82	37,2°	−2,0
14.11.2018	11	153	3,49	34,6°	−1,5	16	119	2,78	33,7°	−1,5
29.11.2018	15	146	3,25	35,6°	+0,5	27	111	2,54	32,5°	−2,25
13.12.2018	14	155	3,51	36,3°	+0,25	16	125	2,84	38,2°	−1,25
17.01.2019	–	–	–	–	–	12	–	–	–	–
06.02.2019	12	148	3,31	35,7°	0,0	26	122	2,76	36,7°	−1,5
08.08.2019	–	–	–	–	–	12	–	–	–	–

Augeninnendruck (*T*), Kammervolumen (*KV*), Vorderkammertiefe (*VKT*), Kammerwinke (*KW*) und Sphäre ermittelt mittels Autorefraktion zu den angegebenen Zeitpunkten

## Diagnose

Diagnostiziert wurde ein chronisches malignes Glaukom nach Kataraktoperation.

## Therapie und Verlauf

Es erfolgte die stationäre Aufnahme zur Verlaufskontrolle und Therapie mit 250 mg Acetazolamid p.o. 3‑mal täglich sowie Atropin 0,5 % Augentropfen (AT) 4‑mal täglich am LA. Unter dieser Therapie sank der IOD bis zum Folgetag auf 8 mm Hg beidseits und blieb bis zur Entlassung 1 Tag später stabil. Außerdem zeigte sich eine deutliche Vertiefung der Vorderkammer. Die Durchführung einer Iridotomie mit dem Versuch einer Eröffnung der vorderen Glaskörpergrenzmembran und eine Vitrektomie wurden diskutiert, aufgrund des guten Ansprechens auf die konservative Therapie jedoch zunächst nicht durchgeführt. Bei einer Kontrolluntersuchung 4 Tage nach Entlassung aus der stationären Behandlung zeigten sich ein normotoner IOD, eine Zunahme des KV und der VKT, eine Vergrößerung des KW (Abb. 1d) und eine Abnahme der Myopie (Tab. 1), sodass eine Reduktion der Acetazolamid-Dosis auf 125 mg 3‑mal täglich unter Fortsetzung der Atropin-Therapie erfolgte. Bei stabilem Befund 8 Tage später wurde die Therapie mit systemischem Acetazolamid über 6 Tage ausgeschlichen. Zusätzlich wurde die Applikation der Atropin 0,5 % AT auf 2‑mal täglich reduziert. Hierunter zeigte sich ein leichter Druckanstieg, sodass schließlich eine Iridotomie mit dem Versuch der Eröffnung des Kapselsacks und der vorderen Glaskörpergrenzmembran durchgeführt und Acetazolamid erneut angesetzt wurde. Nach Ausschleichen des Acetazolamid und 3‑tägiger Therapiekarenz zeigte sich ein IOD von 12/26 mm Hg, sodass die Eröffnung des Kapselsacks und der vorderen Glaskörpergrenzmembran als nicht erfolgreich angesehen werden musste. Bei guter Wirksamkeit des systemischen Carboanhydrasehemmers beschlossen wir einen Therapieversuch mit Brinzolamid AT 2‑mal täglich und Atropin AT zur Nacht unter regelmäßiger ambulanter Druckkontrolle. Sechs Monate später zeigte sich unter dieser Therapie eine stabile Augeninnendrucklage.

## Diskussion

Das maligne Glaukom ist ein schwer zu behandelndes und potenziell zur Erblindung führendes Krankheitsbild. Es wurde erstmals 1869 von von Graefe als seltene postoperative Komplikation mit kompletter Abflachung der Vorderkammer und Erhöhung des Augeninnendrucks beschrieben [3]. Es tritt vorwiegend nach filtrierender Glaukomchirurgie bei vorbestehendem Engwinkelglaukom auf, kann sich prinzipiell jedoch nach jeglichem intraokularem Eingriff wie in diesem Fall nach Phakoemulsifikation und Hinterkammerlinsenimplantation entwickeln [8]. Der Pathomechanismus des malignen Glaukoms ist nicht gänzlich geklärt. Es scheint eine Fehlleitung des Kammerwassers in den Bereich des vorderen Glaskörpers hinter die vordere Glaskörpergrenzmembran zu erfolgen, weshalb auch der Begriff „aqueous misdirection“ für die Erkrankung verwandt wird. Hierdurch steigt der Druck im hinteren Kompartiment des Auges an, und das Iris-Linsen-Diaphragma verlegt sich nach anterior. Dies wiederum verengt den Kammerwinkel und den Kammerwasserabfluss [4]. Die deutsche Bezeichnung ist im Vergleich zum englischen Begriff nicht vom Pathomechanismus abgeleitet und scheint in der Literatur nicht immer gänzlich einheitlich verwendet zu werden. Die Bezeichnung malignes Glaukom wird hierbei nicht mehr nur für die ursprünglich beschriebene komplette Vorderkammeraufhebung, sondern auch für eine nur partielle Abflachung verwendet.

Aufgrund des seltenen Auftretens des malignen Glaukoms finden sich in der Literatur hauptsächlich Fallberichte und -reihen, sodass verschiedene Therapiekonzepte bestehen. Die Therapieoptionen reichen von medikamentöser Therapie über Laseranwendungen mit dem Ziel der Eröffnung der vorderen Glaskörpergrenzmembran bis hin zu intraokularen Eingriffen, in der Regel einer (v. a. vorderen) Vitrektomie [1, 2, 5]. Ziel hierbei ist es, die Fehlleitung des Kammerwassers in den Glaskörper zu verhindern und ein unikamerales System zu schaffen.

Durch Zykloplegie mittels Atropin-Augentropfen und hierdurch herbeigeführter Streckung der Zonula wurde eine Rekonstruktion der Vorderkammer erreicht und die Kammerwasserproduktion durch systemisches Acetazolamid reduziert. Aufgrund der guten Druckeinstellung unter dieser Therapie entschieden wir uns gemeinsam mit der Patientin zunächst für ein möglichst wenig invasives Vorgehen. Die Möglichkeit eines Rezidivs wurde mit der Patientin besprochen. Im mittelfristigen Verlauf blieb der Augeninnendruck unter dauerhafter Lokaltherapie mit Brinzolamid- (2-mal täglich) und Atropin-Augentropfen (1-mal täglich zur Nacht) am LA im Referenzbereich, der Gesichtsfeldbefund war stabil, und der Visus stieg auf 0,63 mit Reduktion der Myopisierung an.

Differenzialdiagnostisch kann in unserem Fall auch diskutiert werden, ob wirklich ein klassisches malignes Glaukom vorlag oder die Vorverlagerung des Iris-Linsen-Diaphragmas nicht auch in einer Schwäche des Zonulaapparates begründet sein könnte. Hierfür gab es klinisch jedoch keine direkten Hinweise. Ein PEX-Syndrom lag nicht vor, auch eine Pseudophakodonesis konnte nicht gesehen werden.

In einer großen Fallserie mit insgesamt 64 Augen konnte in 8 Augen mit medikamentöser und/oder Laserintervention ein Therapieerfolg erzielt werden, der sich bezüglich des Visus, des IOD und der Anzahl an langfristig einzunehmenden augeninnendrucksenkenden Augentropfen nicht von den operierten Augen unterschied [8].

**Figure 4:**
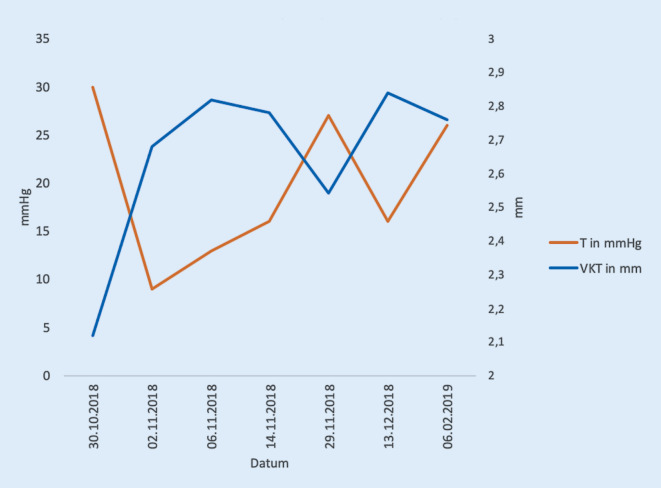

Interessanterweise wird nur selten von einer Myopisierung beim Auftreten eines malignen Glaukoms berichtet [6, 7, 9]. Wie in unserem Fall kann sie jedoch sowohl zur Diagnose als auch zur Kontrolle des Therapieerfolgs beim malignen Glaukom von Bedeutung sein (Abb. 4). Eine Nd:YAG-Iridotomie und eine zentrale Pars-plana-Vitrektomie führen zur Kammerwasserströmung in die VK und somit Rückverlagerung der IOL, ggf. bis zur Hyperopisierung. Außergewöhnlich in unserem Fall war auch die Latenz des Auftretens 7 Jahre nach der Kataraktoperation. Die Latenz zwischen vorangegangener Operation und Auftreten des malignen Glaukoms kann jedoch sehr variabel sein und sollte daher bei den differenzialdiagnostischen Überlegungen bei Tensioerhöhung auch Jahre nach einem intraokularen Eingriff bedacht werden [4].

## Fazit für die Praxis

Eine Myopisierung des betroffenen Auges kann einen Hinweis auf das Vorliegen eines malignen Glaukoms geben.

Die anatomische Rekonstruktion der Vorderkammer, der Augeninnendruck und die Refraktionswerte gehen im Verlauf miteinander einher.

Bei der Vielzahl der therapeutischen Optionen muss jeweils ein individuelles Vorgehen entwickelt werden. Auch eine nichtoperative Therapie kann einen suffizienten und dauerhaften Therapieerfolg erzielen.
